# Diethyl 4,4′-(3,6-dioxaoctane-1,8-diyl­dioxy)dibenzoate

**DOI:** 10.1107/S1600536812004874

**Published:** 2012-02-17

**Authors:** Zhen Ma, Haisha Qin, Gang Lai, Jingjie Fan

**Affiliations:** a School of Chemistry and Chemical Engeneering, Guangxi University, Guangxi 530004, People’s Republic of China

## Abstract

The title compound, C_24_H_30_O_8_, was obtained by reaction of ethyl 4-hy­droxy­benzoate with 1,2-dichloro­ethane. The mol­ecule occupies a crystallographic inversion center, with its central ethyl­ene bridge in an *anti* conformation. The other ethyl­ene bridge has a *gauche* conformation, with the corresponding O—C—C—O torsion angle being 74.2 (1)°. The benzene rings are almost coplanar with the adjacent eth­oxy­carbonyl groups, with an r.m.s. deviation of 0.078 Å.

## Related literature

For the synthesis, structures and applications of diesters, see Hou & Kan (2007 ▶); Tashiro *et al.* (1990 ▶); Zhang *et al.* (2007 ▶). For binding properties and applications of diesters, see: Chen & Liu (2002 ▶). For the synthesis of the title compound, see: Ma & Liu (2002 ▶); Ma & Cao (2011 ▶); Ma & Yang, (2011 ▶). For standard bond lengths, see: Allen *et al.* (1987 ▶).
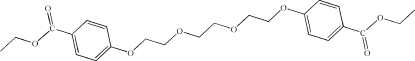


## Experimental

### 

#### Crystal data

C_24_H_30_O_8_
*M* *_r_* = 446.48
Monoclinic, 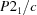

*a* = 9.2471 (17) Å
*b* = 12.530 (2) Å
*c* = 13.275 (2) Å
β = 131.528 (10)°
*V* = 1151.5 (3) Å^3^
*Z* = 2
Mo *K*α radiation
μ = 0.10 mm^−1^
*T* = 298 K
0.46 × 0.41 × 0.39 mm

#### Data collection

Bruker SMART CCD area-detector diffractometer
Absorption correction: multi-scan (*SADABS*; Sheldrick, 1996 ▶) *T*_min_ = 0.957, *T*_max_ = 0.963
16767 measured reflections
5154 independent reflections
2879 reflections with *I* > 2σ(*I*)
*R*_int_ = 0.028

#### Refinement

*R*[*F* ^2^ > 2σ(*F* ^2^)] = 0.051
*wR*(*F* ^2^) = 0.164
*S* = 1.05
5154 reflections
146 parameters
H-atom parameters constrained
Δρ_max_ = 0.34 e Å^−3^
Δρ_min_ = −0.23 e Å^−3^

### 

Data collection: *SMART* (Bruker, 2001 ▶); cell refinement: *SAINT* (Bruker, 2002 ▶); data reduction: *SAINT*; program(s) used to solve structure: *SHELXS97* (Sheldrick, 2008 ▶); program(s) used to refine structure: *SHELXL97* (Sheldrick, 2008 ▶); molecular graphics: *SHELXTL* (Sheldrick, 2008 ▶); software used to prepare material for publication: *SHELXL97*.

## Supplementary Material

Crystal structure: contains datablock(s) I, global. DOI: 10.1107/S1600536812004874/ld2043sup1.cif

Structure factors: contains datablock(s) I. DOI: 10.1107/S1600536812004874/ld2043Isup2.hkl

Supplementary material file. DOI: 10.1107/S1600536812004874/ld2043Isup3.cml

Additional supplementary materials:  crystallographic information; 3D view; checkCIF report

## Figures and Tables

**Fig. 1.**
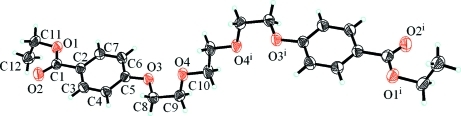
The molecular structure of the title compound with the atom numbering scheme. Displacement ellipsoids are drawn at the 50% probability level. H atoms are presented as small spheres of arbitrary radius.

## supplementary crystallographic information

### Comment

This paper represents a part of our continuing study on the synthesis and structural characterization of dialdehydes and diesters (Ma & Liu, 2002; Ma & Cao, 2011a; Ma & Yang, 2011b). We are interested in utilization of these compounds as precusors for the synthesis of macrocyclic or macrobicyclic compounds, and for manufacturing of different coordination topologies (Chen & Liu 2002) for various applications (Hou & Kan, 2007; Tashiro *et al.*, 1990; Zhang *et al.*, 2007). We report here the X-ray structure of a new diester compound (Fig. 1) along with elemental analysis and IR data. All bond lengths are within normal ranges (Allen *et al.*, 1987). The two aromatic rings are parallel to each other because of the molecular symmetry.

### Experimental

The title compound was obtained by the reaction of ethyl 4-hydroxybenzoate with 1,2-bis(2-chloro-ethoxy)ethane in *N*,*N*'-dimethylformamide (DMF) in the presence of K_2_CO_3_ according to a reported procedure (Ma & Liu, 2002; Ma & Cao, 2011; Ma & Yang, 2011). In a 100 cm^3^ flask fitted with a funnel, ethyl 4-hydroxybenzoate (8.3 g, 50 m*M*) and potassium carbonate (14 g, 100 m*M*) were mixed in 50 cm^3^ of DMF. A stoichiometric quantity of 1,2-bis(2-chloro-ethoxy)ethane (4.7 g, 25 m*M*) dissolved in 20 cm^3^ of DMF has been added dropwise to this solution for a period of one hour with continuous stirring. The mixture was then stirred for 24 h at 353 K. The solution was concentrated under reduced pressure and the white solid formed by adding a large quantity of water (200 cm^3^) was filtered off and recrystallized from ethanol and decolored with activated carbon. A colorless solid was obtained (Yield 80 %, m.p: 337–339 K). Anal. Calcd. for [C_24_H_30_O_8_](C_2_H_6_O)_1/2_ (%): C, 63.95; H, 7.08; found: C, 64.23; H, 6.87; IR (KBr), (cm^-1^): 2938 (w), 1707, (s, C=O), 1606, 1513, 1466 (s, C=C of aryl), 1281, 1253, 1175, 1131, 1106 (CH_2_—O—CH_2_), 1066, 1048, 1014, 929-653, (Ar—H). Slow evaporation of a solution of the title compound in ethanol and dichloromethane (1:1) led to the formation of colorless crystals, which were suitable for X-ray characterization.

### Refinement

All H atoms were positioned geometrically and refined using riding and rotating model with C—H = 0.93 - 0.97 Å, with *U*_iso_(H) = 1.5 times *U*_eq_(C) for methyl H atoms and *U*_iso_(H) = 1.2 times *U*_eq_(C) for all other H atoms.

### Crystal data

**Table:**

C_24_H_30_O_8_	*F*(000) = 476
*M**_r_* = 446.48	*D*_x_ = 1.288 Mg m^−^^3^
Monoclinic, *P*2_1_/*c*	Mo *K*α radiation, λ = 0.71073 Å
Hall symbol: -P 2ybc	Cell parameters from 16767 reflections
*a* = 9.2471 (17) Å	θ = 2.6–35.5°
*b* = 12.530 (2) Å	µ = 0.10 mm^−^^1^
*c* = 13.275 (2) Å	*T* = 298 K
β = 131.528 (10)°	Prism, colorless
*V* = 1151.5 (3) Å^3^	0.46 × 0.41 × 0.39 mm
*Z* = 2	

### Data collection

**Table:**

Bruker SMART CCD area-detector diffractometer	5154 independent reflections
Radiation source: fine-focus sealed tube	2879 reflections with *I* > 2σ(*I*)
Graphite Monochromator monochromator	*R*_int_ = 0.028
Detector resolution: 0 pixels mm^-1^	θ_max_ = 35.5°, θ_min_ = 2.6°
phi and ω scans	*h* = −15→15
Absorption correction: multi-scan (*SADABS*; Sheldrick, 1996)	*k* = −20→20
*T*_min_ = 0.957, *T*_max_ = 0.963	*l* = −21→20
16767 measured reflections	

### Refinement

**Table:**

Refinement on *F*^2^	Primary atom site location: structure-invariant direct methods
Least-squares matrix: full	Secondary atom site location: difference Fourier map
*R*[*F*^2^ > 2σ(*F*^2^)] = 0.051	Hydrogen site location: inferred from neighbouring sites
*wR*(*F*^2^) = 0.164	H-atom parameters constrained
*S* = 1.05	*w* = 1/[σ^2^(*F*_o_^2^) + (0.0674*P*)^2^ + 0.1605*P*] where *P* = (*F*_o_^2^ + 2*F*_c_^2^)/3
5154 reflections	(Δ/σ)_max_ < 0.001
146 parameters	Δρ_max_ = 0.34 e Å^−^^3^
0 restraints	Δρ_min_ = −0.23 e Å^−^^3^

### Special details

**Table:**

Geometry. All esds (except the esd in the dihedral angle between two l.s. planes) are estimated using the full covariance matrix. The cell esds are taken into account individually in the estimation of esds in distances, angles and torsion angles; correlations between esds in cell parameters are only used when they are defined by crystal symmetry. An approximate (isotropic) treatment of cell esds is used for estimating esds involving l.s. planes.
Refinement. Refinement of F^2^ against ALL reflections. The weighted R-factor wR and goodness of fit S are based on F^2^, conventional R-factors R are based on F, with F set to zero for negative F^2^. The threshold expression of F^2^ > 2sigma(F^2^) is used only for calculating R-factors(gt) etc. and is not relevant to the choice of reflections for refinement. R-factors based on F^2^ are statistically about twice as large as those based on F, and R- factors based on ALL data will be even larger.

### Fractional atomic coordinates and isotropic or equivalent isotropic displacement parameters (Å^2^)

**Table:**

	*x*	*y*	*z*	*U*_iso_*/*U*_eq_	
O1	−0.82218 (13)	0.56947 (7)	−0.25960 (9)	0.0452 (2)	
O2	−0.61141 (15)	0.54988 (8)	−0.28929 (11)	0.0530 (3)	
O3	−0.42322 (12)	0.15018 (7)	0.07878 (8)	0.0403 (2)	
O4	−0.11518 (12)	0.04803 (7)	0.33477 (8)	0.0406 (2)	
C1	−0.68021 (16)	0.51813 (9)	−0.24305 (12)	0.0374 (2)	
C2	−0.61826 (15)	0.41940 (9)	−0.16212 (11)	0.0341 (2)	
C3	−0.68284 (16)	0.39335 (10)	−0.09580 (12)	0.0379 (2)	
H3A	−0.7724	0.4370	−0.1051	0.045*	
C4	−0.61502 (17)	0.30326 (10)	−0.01627 (12)	0.0392 (3)	
H4A	−0.6582	0.2868	0.0283	0.047*	
C5	−0.48191 (15)	0.23687 (9)	−0.00255 (11)	0.0332 (2)	
C6	−0.41928 (18)	0.26088 (10)	−0.07013 (13)	0.0418 (3)	
H6A	−0.3329	0.2159	−0.0631	0.050*	
C7	−0.48635 (18)	0.35259 (10)	−0.14845 (13)	0.0421 (3)	
H7A	−0.4423	0.3695	−0.1923	0.051*	
C8	−0.27474 (17)	0.08362 (10)	0.10606 (11)	0.0384 (2)	
H8A	−0.1616	0.1261	0.1424	0.046*	
H8B	−0.3199	0.0500	0.0237	0.046*	
C9	−0.22479 (18)	0.00033 (10)	0.20575 (11)	0.0399 (3)	
H9A	−0.3420	−0.0305	0.1791	0.048*	
H9B	−0.1505	−0.0563	0.2086	0.048*	
C10	−0.06051 (19)	−0.02821 (10)	0.43318 (12)	0.0441 (3)	
H10A	0.0132	−0.0852	0.4361	0.053*	
H10B	−0.1743	−0.0590	0.4119	0.053*	
C11	−0.8867 (2)	0.67038 (10)	−0.33238 (13)	0.0460 (3)	
H1	−0.7765	0.7084	−0.3088	0.055*	
H2	−0.9433	0.7141	−0.3060	0.055*	
C12	−1.0324 (2)	0.65276 (11)	−0.48130 (15)	0.0538 (3)	
H3	−1.0756	0.7204	−0.5266	0.081*	
H4	−1.1405	0.6140	−0.5046	0.081*	
H5	−0.9745	0.6126	−0.5082	0.081*	

### Atomic displacement parameters (Å^2^)

**Table:**

	*U*^11^	*U*^22^	*U*^33^	*U*^12^	*U*^13^	*U*^23^
O1	0.0496 (5)	0.0386 (4)	0.0516 (5)	0.0099 (4)	0.0354 (5)	0.0108 (4)
O2	0.0629 (6)	0.0488 (5)	0.0639 (6)	0.0099 (4)	0.0490 (6)	0.0171 (4)
O3	0.0443 (4)	0.0400 (4)	0.0390 (4)	0.0081 (3)	0.0286 (4)	0.0109 (3)
O4	0.0449 (4)	0.0384 (4)	0.0283 (4)	−0.0018 (3)	0.0200 (4)	0.0060 (3)
C1	0.0387 (5)	0.0340 (5)	0.0365 (6)	0.0004 (4)	0.0237 (5)	0.0012 (4)
C2	0.0342 (5)	0.0336 (5)	0.0318 (5)	−0.0002 (4)	0.0207 (5)	0.0015 (4)
C3	0.0350 (5)	0.0391 (6)	0.0418 (6)	0.0050 (4)	0.0265 (5)	0.0048 (5)
C4	0.0398 (6)	0.0429 (6)	0.0419 (6)	0.0015 (5)	0.0301 (5)	0.0058 (5)
C5	0.0337 (5)	0.0334 (5)	0.0279 (5)	−0.0001 (4)	0.0184 (4)	0.0018 (4)
C6	0.0493 (6)	0.0420 (6)	0.0451 (6)	0.0120 (5)	0.0359 (6)	0.0090 (5)
C7	0.0505 (7)	0.0439 (6)	0.0438 (6)	0.0073 (5)	0.0362 (6)	0.0085 (5)
C8	0.0412 (6)	0.0407 (6)	0.0307 (5)	0.0063 (5)	0.0227 (5)	0.0054 (4)
C9	0.0435 (6)	0.0361 (6)	0.0325 (5)	0.0030 (5)	0.0220 (5)	0.0029 (4)
C10	0.0472 (6)	0.0412 (6)	0.0331 (6)	−0.0003 (5)	0.0220 (5)	0.0096 (5)
C11	0.0531 (7)	0.0315 (6)	0.0506 (7)	0.0063 (5)	0.0333 (6)	0.0033 (5)
C12	0.0563 (8)	0.0441 (7)	0.0519 (8)	0.0086 (6)	0.0320 (7)	0.0073 (6)

### Geometric parameters (Å, º)

**Table:**

O1—C1	1.3428 (14)	C6—H6A	0.9300
O1—C11	1.4573 (15)	C7—H7A	0.9300
O2—C1	1.2072 (14)	C8—C9	1.4978 (16)
O3—C5	1.3650 (13)	C8—H8A	0.9700
O3—C8	1.4327 (14)	C8—H8B	0.9700
O4—C10	1.4140 (13)	C9—H9A	0.9700
O4—C9	1.4199 (14)	C9—H9B	0.9700
C1—C2	1.4821 (15)	C10—C10^i^	1.506 (3)
C2—C7	1.3886 (16)	C10—H10A	0.9700
C2—C3	1.3912 (16)	C10—H10B	0.9700
C3—C4	1.3800 (16)	C11—C12	1.497 (2)
C3—H3A	0.9300	C11—H1	0.9700
C4—C5	1.3939 (16)	C11—H2	0.9700
C4—H4A	0.9300	C12—H3	0.9600
C5—C6	1.3856 (15)	C12—H4	0.9600
C6—C7	1.3896 (17)	C12—H5	0.9600
			
C1—O1—C11	116.75 (10)	O3—C8—H8B	110.1
C5—O3—C8	118.36 (9)	C9—C8—H8B	110.1
C10—O4—C9	111.23 (9)	H8A—C8—H8B	108.4
O2—C1—O1	123.13 (11)	O4—C9—C8	109.14 (10)
O2—C1—C2	124.26 (11)	O4—C9—H9A	109.9
O1—C1—C2	112.62 (10)	C8—C9—H9A	109.9
C7—C2—C3	118.97 (10)	O4—C9—H9B	109.9
C7—C2—C1	118.80 (10)	C8—C9—H9B	109.9
C3—C2—C1	122.19 (10)	H9A—C9—H9B	108.3
C4—C3—C2	120.53 (10)	O4—C10—C10^i^	107.61 (12)
C4—C3—H3A	119.7	O4—C10—H10A	110.2
C2—C3—H3A	119.7	C10^i^—C10—H10A	110.2
C3—C4—C5	120.15 (10)	O4—C10—H10B	110.2
C3—C4—H4A	119.9	C10^i^—C10—H10B	110.2
C5—C4—H4A	119.9	H10A—C10—H10B	108.5
O3—C5—C6	124.56 (10)	O1—C11—C12	111.23 (11)
O3—C5—C4	115.59 (9)	O1—C11—H1	109.4
C6—C5—C4	119.85 (10)	C12—C11—H1	109.4
C5—C6—C7	119.55 (10)	O1—C11—H2	109.4
C5—C6—H6A	120.2	C12—C11—H2	109.4
C7—C6—H6A	120.2	H1—C11—H2	108.0
C2—C7—C6	120.93 (10)	C11—C12—H3	109.5
C2—C7—H7A	119.5	C11—C12—H4	109.5
C6—C7—H7A	119.5	H3—C12—H4	109.5
O3—C8—C9	108.14 (9)	C11—C12—H5	109.5
O3—C8—H8A	110.1	H3—C12—H5	109.5
C9—C8—H8A	110.1	H4—C12—H5	109.5
			
C11—O1—C1—O2	−2.72 (18)	C3—C4—C5—C6	−0.70 (18)
C11—O1—C1—C2	177.11 (10)	O3—C5—C6—C7	−178.83 (11)
O2—C1—C2—C7	−6.63 (18)	C4—C5—C6—C7	1.60 (19)
O1—C1—C2—C7	173.54 (10)	C3—C2—C7—C6	0.10 (19)
O2—C1—C2—C3	171.16 (12)	C1—C2—C7—C6	177.96 (11)
O1—C1—C2—C3	−8.67 (16)	C5—C6—C7—C2	−1.3 (2)
C7—C2—C3—C4	0.82 (18)	C5—O3—C8—C9	175.32 (9)
C1—C2—C3—C4	−176.96 (11)	C10—O4—C9—C8	−178.60 (10)
C2—C3—C4—C5	−0.53 (18)	O3—C8—C9—O4	−74.22 (12)
C8—O3—C5—C6	5.50 (17)	C9—O4—C10—C10^i^	177.97 (13)
C8—O3—C5—C4	−174.91 (10)	C1—O1—C11—C12	83.70 (14)
C3—C4—C5—O3	179.70 (10)		

Symmetry code: (i) −*x*, −*y*, −*z*+1.
